# Differential packaging of inflammatory cytokines/ chemokines and oxidative stress modulators in U937 and U1 macrophages-derived extracellular vesicles upon exposure to tobacco constituents

**DOI:** 10.1371/journal.pone.0233054

**Published:** 2020-05-20

**Authors:** Sanjana Haque, Sunitha Kodidela, Namita Sinha, Prashant Kumar, Theodore J. Cory, Santosh Kumar

**Affiliations:** 1 Department of Pharmaceutical Sciences, College of Pharmacy, University of Tennessee Health Science Center, Memphis, TN, United States of America; 2 Division of Pediatric Nephrology, Le Bonheur Children's Hospital, University of Tennessee Health Science Center, Memphis, TN, United States of America; 3 Department of Clinical Pharmacy and Translational Sciences, College of Pharmacy, University of Tennessee Health Science Center, Memphis, TN, United States of America; Harvard Medical School, UNITED STATES

## Abstract

Smoking, which is highly prevalent in HIV-infected populations, has been shown to exacerbate HIV replication, in part via the cytochrome P450 (CYP)-induced oxidative stress pathway. Recently, we have shown that extracellular vesicles (EVs), derived from tobacco- and/or HIV-exposed macrophages, alter HIV replication in macrophages by cell-cell interactions. We hypothesize that cigarette smoke condensate (CSC) and/or HIV-exposed macrophage-derived EVs carry relatively high levels of pro-oxidant and pro-inflammatory cargos and/or low levels of antioxidant and anti-inflammatory cargos, which are key mediators for HIV pathogenesis. Therefore, in this study, we investigated differential packaging of pro- and anti-inflammatory cytokines/chemokines and pro- and anti-oxidant contents in EVs after CSC exposure to myeloid cells (uninfected U937 and HIV-infected U1 cells). Our results showed that relatively long to short exposures with CSC increased the expression of cytokines in EVs isolated from HIV-infected U1 macrophages. Importantly, pro-inflammatory cytokines, especially IL-6, were highly packaged in EVs isolated from HIV-infected U1 macrophages upon both long and short-term CSC exposures. In general, anti-inflammatory cytokines, particularly IL-10, had a lower packaging in EVs, while packaging of chemokines was mostly increased in EVs upon CSC exposure in both HIV-infected U1 and uninfected U937 macrophages. Moreover, we observed higher expression of CYPs (1A1 and 1B1) and lower expression of antioxidant enzymes (SOD-1 and catalase) in EVs from HIV-infected U1 macrophages than in uninfected U937 macrophages. Together, they are expected to increase oxidative stress factors in EVs derived from HIV-infected U1 cells. Taken together, our results suggest packaging of increased level of oxidative stress and inflammatory elements in the EVs upon exposure to tobacco constituents and/or HIV to myeloid cells, which would ultimately enhance HIV replication in macrophages via cell-cell interactions.

## Introduction

The prevalence of smoking is higher in people living with HIV/AIDS (PLWHA) (40–70%) vs. the general population (15–25%) [1, 2]. Further, smoking has been shown to exacerbate HIV pathogenesis and its related comorbidities by dysregulating the cytokine and chemokine expression as well as increasing oxidative stress [3, 4]. Cytokines and chemokines play a critical role in the immune system by providing a precise control mechanism in the migration and position of immune cells [5]. Imbalances in the levels of cytokines and chemokines during HIV infection vary greatly depending upon the stages of infection [6]. Our earlier studies have also shown dysregulation of cytokines and chemokines in HIV-infected smokers and non-smokers [4, 7]. In addition, it is reported that cytokines have a significant effect on the mRNA and protein expressions of cytochrome P450 (CYP) enzymes in peripheral blood mononuclear cells (PBMCs) [8]. It has been suggested that the regulation of CYP expression by cytokines is highly variable, which could potentially vary with different inflammatory disease conditions [9, 10]. Further, we have shown that CYP enzymes responsible for the metabolism of smoking constituents cause oxidative stress and DNA damage, ultimately leading to the progression of HIV replication in macrophages via an oxidative stress pathway [7, 11, 12]. Macrophages serve as one of the major reservoirs for HIV, contributing to HIV pathogenesis and disease progression [13]. Literatures and our own studies have, in part, established the role of cellular oxidative stress in HIV pathogenesis by altering the pro- and anti-inflammatory and pro- and antioxidant factors in monocytes and macrophages upon exposure to cigarette smoke [14, 15]. However, how these cellular changes contribute to exacerbated HIV pathogenesis in distant/other cells, especially in the presence of smoking constituents, needs to be investigated.

Extracellular vesicles (EVs) are a promising group of biological molecules, which are shown to play an important role in cell-cell communication by transferring various biological cargo to recipient cells [16–19]. When cells undergo stress in response to an external stimulus, the production of either toxic or protective components is increased. EVs can subsequently package these substances, which reflect the pathological condition of the parent cells upon external stimulation. We have demonstrated that EVs can be either protective or toxic to recipient cells [20]. Specifically, EVs originating from HIV-uninfected monocytes package protective elements, whereas EVs from HIV-infected cells lose this protective capacity. Our next step is to find the components packaged in EVs that might be responsible for such effects. Therefore, in this study, we aimed to investigate the packaging of inflammatory and oxidative stress modulators in EVs. In our previous study, we measured the levels of cytokines and chemokines in EVs derived from HIV-positive drug abusers. We observed differential packaging of these agents in EVs derived from HIV-infected and/or drugs of abuse-exposed populations [4]. In this study, we aimed to measure cytokines/chemokines, as well as oxidative stress-regulating elements, in EVs upon exposure to cigarette smoke condensate (CSC) to myeloid cells (uninfected U937 and HIV-infected U1 cells). We hypothesize that CSC and/or HIV-infected U1 macrophage-derived EVs carry relatively high levels of key mediators of HIV pathogenesis, pro-inflammatory and pro-oxidant cargos and/or low levels of anti-inflammatory and antioxidant cargos compared with uninfected U937 macrophages.

## Materials and methods

### Cell culture

Monocyte derived macrophages (MDM) were used to isolate extracellular vesicles. Two myeloid cell lines- U937 cells (ATCC, Manassas, VA) and latently HIV-infected U1 cells (NIH AIDS Reagent Program, Germantown, MD) were differentiated into macrophages. U937 and U1 are model cell lines, which have been used extensively by our group and by many other researchers to study the effects of drug abuse, including tobacco smoking in the setting of HIV [15, 20–23]. U1 is a subclone of the U937 cell line, which is chronically infected with HIV. The data obtained from these cell lines have been consistently verified and strongly correlated with primary macrophages [23, 24]. Since U1 are the progenitor cells derived from U937 upon HIV infection, and our experimental protocols for obtaining data from both cell lines are identical, we have compared the data obtained between these two cells. Multiple studies have also compared similar outcomes between these two cells lines [22, 25]. Cells were maintained in Roswell Park Memorial Institute (RPMI) 1640 media, supplemented with fetal bovine serum (FBS), sodium bicarbonate, L-glutamine, and gentamicin or penicillin/streptomycin. For differentiation, 0.8 million cells/well were seeded in 6-well plates with RPMI media containing 80nM phorbol 12-myristate 13-acetate (PMA) and kept in an incubator (37°C, 5% CO_2_) for three days. Afterwards, media and non-adherent cells were removed from the wells, washed with phosphate buffer saline (PBS) followed by addition of exosome-depleted FBS (Exo-FBS)-containing RPMI media. The cells were treated with 10 μg/ml of cigarette smoke condensate (CSC) (Murty Pharmaceuticals, Inc, KY) for at least 4 days (short-term exposure) or 6–8 days (long-term exposure), as described previously [20]. Treating cells for 4 days allows optimum time for EVs to capture the contents within cells and get released into the media. With longer exposure, the CSC treatment is observed to be toxic to the cells after 6 and 8 days in U1 and U937 cells, respectively. Therefore, our long-term exposure was defined by 6–8 days exposure. After the designated treatment period, the cells were harvested, and the supernatant was collected for EV isolation.

### Isolation of EVs

To isolate EVs from cells, we used the Invitrogen-Total Exosome Isolation (from cell culture media) kit (Life Technologies, NY), following the kit protocol. Earlier, we established the isolation of EVs using the gold standard ultracentrifugation method, as well as EV characterizations by measuring various EV proteins, enzyme markers and by utilizing transmission electron microscopy, as per ISEV guidelines [20]. Our previous studies have demonstrated that EVs isolated using the commercial kit can retain their purity and properties needed for our study, similar to the classical ultracentrifugation procedure [20, 26].

### Characterization of EVs

The isolated EVs were evaluated by tunable resistive pulse sensing (TRPS), with the help of qNano gold (Izon Science, Christchurch, New Zealand). Initially, the instrument was calibrated with polystyrene beads (CPC100), using a final dilution of 1:500. 35μl of each sample was analyzed using a polyurethane nanopore (NP150, Izon), with a stretch of 47.01nm and blockade voltage of 0.30mV. The standard and EV samples were allowed to pass through the nanopore at 15mbar pressure. A minimum of 500 particles was counted for each run. Data processing and analysis were performed using the Izon control suite v3.3 as per the manufacturer instructions. Zeta potential of the isolated EVs was measured using the Zetasizer Nano-ZS (Malvern Instruments Inc, Malvern, UK) as described earlier [20]. Briefly, EV pellets were resuspended in 0.2μm filtered DI water with addition of 100μl 0.2μl 10XPBS. The EV sample was then subjected to dynamic light scattering in the Zetasizer. EV protein quantification was performed using Pierce^™^ BCA protein assay (ThermoFisher Scientific, Grand Island, NY).

### Cytokine analysis

For cytokine analysis, we used freshly isolated EVs from 500μl media and respective cell media. The levels of selected cytokines and chemokines, such as pro-inflammatory cytokines: IL-6, IL-8, IL-1β, and TNF-α; anti-inflammatory cytokines: IL-1ra and IL-10; chemokines: MCP-1 and RANTES were measured using ProcartaPlex^™^ Multiplex Immunoassay (Invitrogen, ThermoFisher Scientific, NY). The EV pellets were resuspended in 50μl Universal Assay buffer, and 50μl cell media was taken directly from the respective well. Following the kit protocol, the samples, standards, and magnetic beads were added to the 96-well ELISA plate and incubated (shaking at room temperature) for 1 hour. The beads were washed, followed by addition of the detection antibody, streptavidin-PE, and reading buffer, with subsequent washing off of reagents. The final protein concentration was measured using the Luminex 200^TM^ system, and the data were analyzed by xPONENT^®^ software. Next, we calculated the percent of cytokines packaged in EVs. We analyzed the relative packaging of cytokines in EVs compared with cytokines in the media from which the EVs originated. The “Percent of EV packaging” refers to the percentage of cytokines packaged in the EVs compared to the cytokines present in the media. We calculated the percentage using the following equation:

PercentofEVpackaging=(ConcentrationofcytokinesintheEVs÷Concentrationofcytokinesinthemedia)×100

### Specific protein analysis by western blot

We isolated EVs from either 500 μl or 1000 μl cell media (as required for a better protein band) from U937 and U1 macrophages treated with 10 μg/ml CSC for short and long-term exposures, and we checked the following protein expressions: CD63, CD81, and CD9 (EV markers), CYP1B1, CYP1A1, and CYP3A4 (oxidative stress-inducing enzymes), SOD-1 and catalase (antioxidant enzymes (AOEs)) with western blotting. The Pierce^™^ BCA protein assay (ThermoFisher Scientific, Grand Island, NY) was used to quantify the protein amount. Equal amounts of protein were loaded in each well of a 10% poly acrylamide gel. Following standard protocol, the gel was run for an hour at 150V to separate the proteins based on their molecular weight. Next, the protein bands were transferred from gel to a polyvinyl fluoride membrane by running the gel and membrane sandwich at 0.35 Amp for 1.5 hour. After that, the membrane was blocked with Li-Cor blocking buffer (LI-COR Biosciences, Lincoln, NE) for 1 hour to prevent any non-specific protein binding. The membrane was incubated overnight at 4°C with target primary antibodies (CD63 rabbit Mab, 1:300 dilution, Proteintech, catalog #25682-1-AP; CD81 rabbit Mab 1:100 dilution, Santa Cruz Biotechnology, catalog #sc-9158; CD9 mouse Mab, 1:100 dilution, Proteintech catalog # 60232-1-1g; CYP1B1 rabbit Mab, 1:200 dilution, Santa Cruz Biotechnology, catalog # sc-32882; CYP1A1 rabbit Mab, 1:200 dilution, Abcam, catalog # ab3568; CYP3A4 mouse Mab, 1:100 dilution, Santa Cruz Biotechnology, catalog # sc-53850; SOD1 mouse Mab, 1:200 dilution, Santa Cruz Biotechnology, catalog #sc-101523; Catalase mouse Mab, 1:100 dilution, Santa Cruz Biotechnology, catalog # sc-365738). The next day, the membrane was washed with 0.2% Tween-20-containing PBS and incubated with its respective secondary antibody at room temperature for 1 hour, protected from light. The membrane was again washed with 0.2% Tween-20-containing PBS and the blot was scanned using the Li-Cor Scanner (LI-COR Biosciences) with Image Studio Lite version 4.0.

### Statistical analysis

Mean ± SEM was calculated and compared to the control group. One-way ANOVA with Tukey’s multiple comparison test was used to calculate the statistical significance. All the statistical calculations were performed using GraphPad Prism 7.

## Results

### Physical characterization of EVs

**Fig 1:**
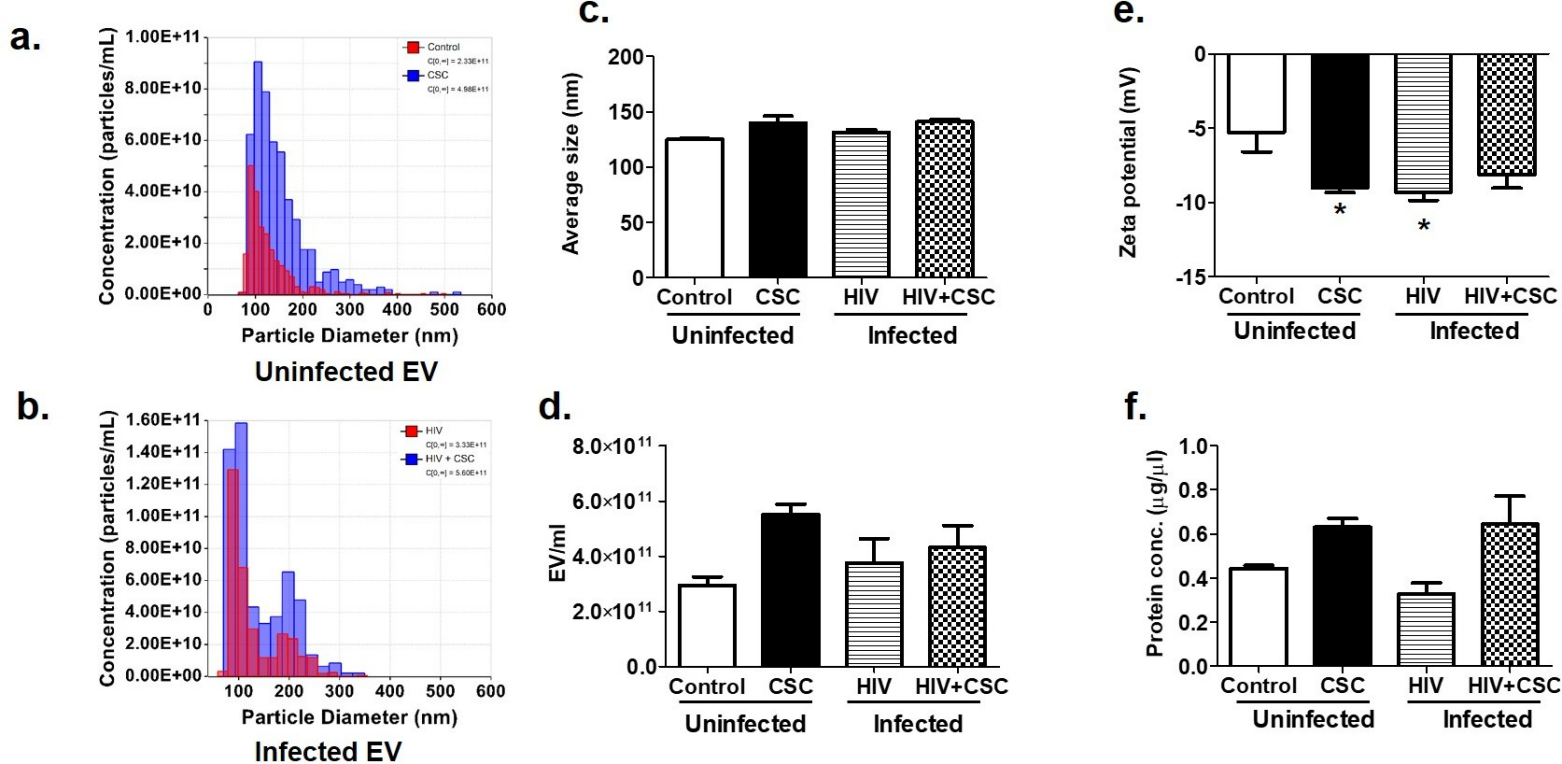
Physical characteristics of Extracellular Vesicles (EV) collected from U937 (uninfected) and U1 (HIV-infected) macrophages treated with CSC. a. Representative diagram of number of EV/ml for uninfected EV. b. Representative diagram of number of EVs/ml for infected EV. c. Average size of EV. d. Number of EV/ml. e. Zeta potential of EV. Bars represent data from n = 3 replicates. *p<0.05 compared to control. f. Total protein concentration of EV. Bars represent data from n = 3 replicates (except the HIV group, which is n = 2).

Physical characteristics of EVs in terms of size, number of EVs, zeta potential, and protein concentration were estimated (Fig 1). The average size of EVs from uninfected U937 and HIV-infected U1 cells was similar, in the presence or absence of CSC (Fig 1A, 1B and 1C). Although not statistically significant, the number of EVs per ml trended upward in the presence of such stressors as CSC and HIV (Fig 1D). Zeta potentials ranged between -5mV to -10mV, which is optimum for EVs (Fig 1E). Next, we measured the total protein concentration in EVs (Fig 1F). We observed that protein concentration gradually increased from the uninfected control group to the uninfected CSC-treated group, with a similar trend noted in the HIV-infected groups (Fig 1F).

### Effect of short-term CSC exposure on cytokines and chemokines

#### Pro-inflammatory cytokines

**Fig 2:**
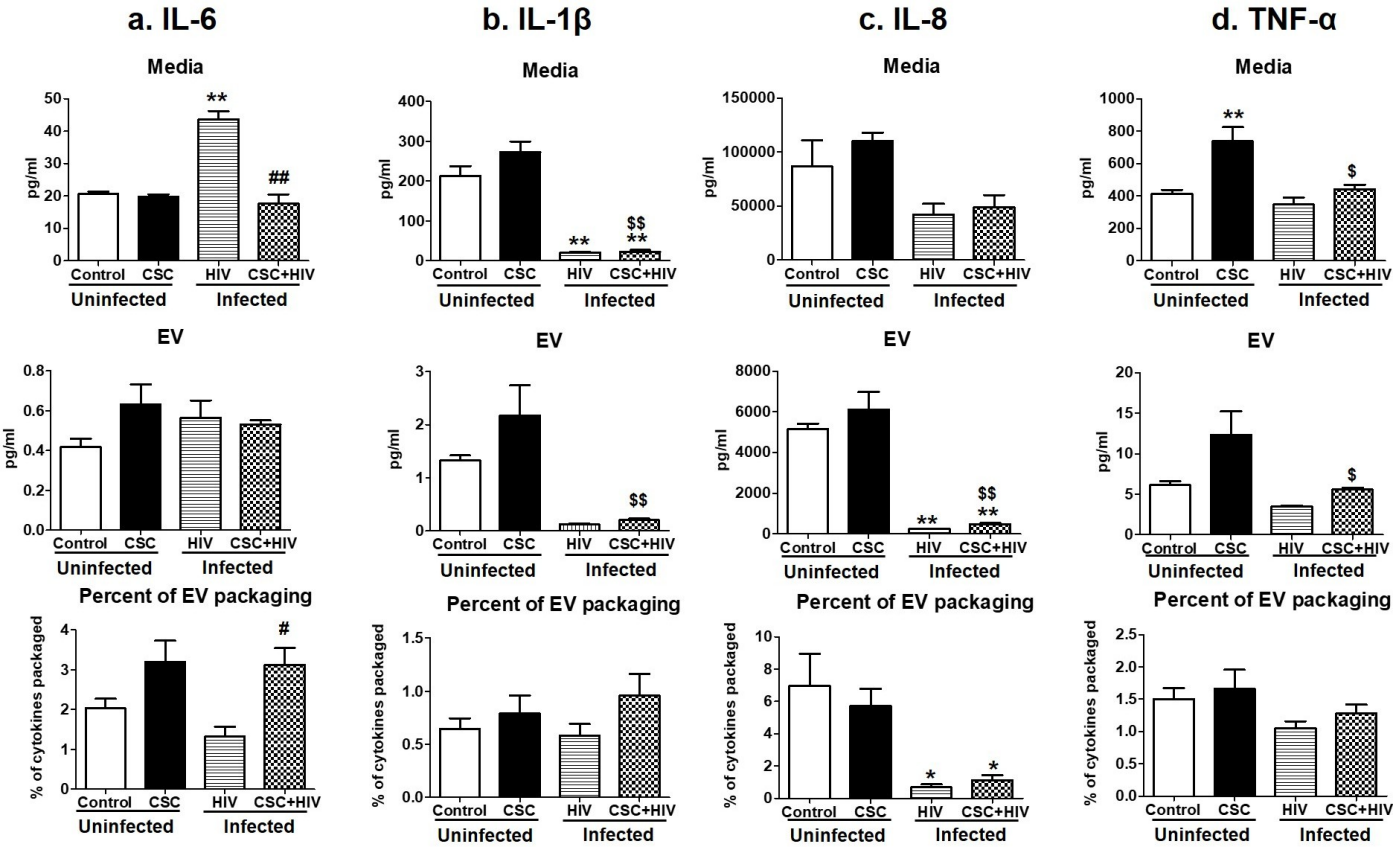
Pro-inflammatory cytokines in media and Extracellular Vesicles (EV) isolated from U937 (uninfected) and U1 (HIV-infected) macrophages treated with CSC short-term. a. Concentration of IL-6 in media, EVs, and percent of cytokine packaged in EV. b. Concentration of IL-1β in media, EV, and percent of cytokine packaged in EV. c. Concentration of IL-8 in media, EV, and percent of cytokine packaged in EV. d. Concentration of TNF-α in media, EV, and percent of cytokine packaged in EV. *p<0.05, **p<0.001 compared to control; #p<0.05, ##p<0.001 compared to HIV. $p<0.05 and $ $p<0.001 compared to CSC. Bars represent data from n = 3 replicates. Statistical analysis performed: One-way ANOVA with Tukey’s Multiple comparison test.

Pro-inflammatory cytokines such as IL-6, IL-1β, IL-8, and TNF-α levels were measured in uninfected and HIV-infected macrophage-derived EVs treated with CSC (Fig 2A–2D). In general, the cytokine levels increased after CSC exposure, both in the media and in EVs from HIV-uninfected U937 cells compared to their respective controls (except IL-6). In the case of HIV-infected U1 macrophage-derived EVs, IL-6 showed an upward trend. Conversely, IL-1β, IL-8 and TNF-α showed a downward trend, both in the media and EVs after CSC treatment. The percentage of cytokines packaged in EVs was low, ranging between 0.05%-6.5% of the total cytokines present in media. Further, the IL-6 level in the media of HIV-infected U1 cells was significantly higher than in the media of uninfected U937 cells. However, in the presence of CSC, IL-6 concentration decreased significantly in the media of HIV-infected U1 cells compared to the media of HIV-infected U1 cells without CSC treatment. EV packaging might be the contributor to this change, which showed a significant rise in cytokine concentration within EVs (Fig 2A). About 3% of total IL-6 was packaged in HIV-infected U1 macrophage-derived EVs upon exposure to CSC, which is higher than in control EVs from U937 cells (Fig 2A). IL-1β also demonstrated higher EV packaging after exposure to CSC, both in the absence and presence of HIV infection (Fig 2B).

#### Anti-inflammatory cytokines

**Fig 3:**
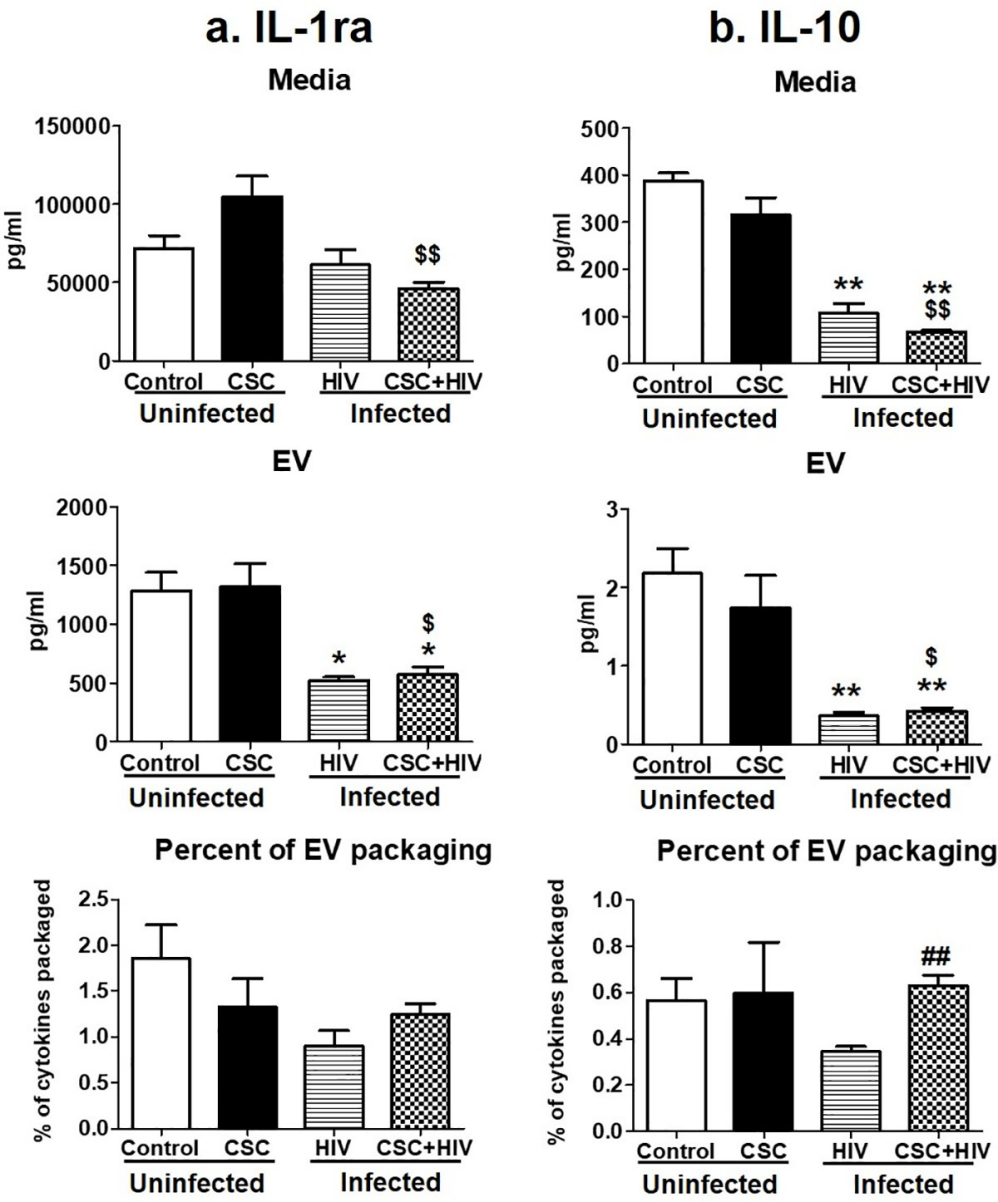
Anti-inflammatory cytokines in media and Extracellular Vesicles (EV) isolated from U937 (uninfected) and U1 (HIV-infected) macrophages treated with CSC short-term. a. Concentration of IL-1ra in media, EV, and percent of cytokine packaged in EV. b. Concentration of IL-10 in media, EV, and percent of cytokine packaged in EV. *p<0.05, **p<0.001 compared to control; #p<0.05, ##p<0.001 compared to HIV. $p<0.05 and $ $p<0.001 compared to CSC. Bars represent data from n = 3 replicates. Statistical analysis performed: One-way ANOVA with Tukey’s Multiple comparison test.

We measured the concentration of two anti-inflammatory cytokines, IL-1ra and IL-10, in the same experimental setting (Fig 3A and 3B). CSC mostly reduced cytokines in the media and in EVs from uninfected U937 and HIV-infected U1 cells (except IL-1ra in CSC treated media and EVs from uninfected cells). However, there was a slight increase in the EV packaging of IL-1ra and IL-10 upon exposure to CSC and in the presence of HIV-infection, compared to their EV packaging without CSC treatment (Fig 3A). Moreover, the packaging of IL-10 in EVs was significantly high (Fig 3B) in CSC-treated HIV-infected U1 cells derived EVs, when compared to CSC treated uninfected U937 cell derived EVs.

#### Chemokines

**Fig 4:**
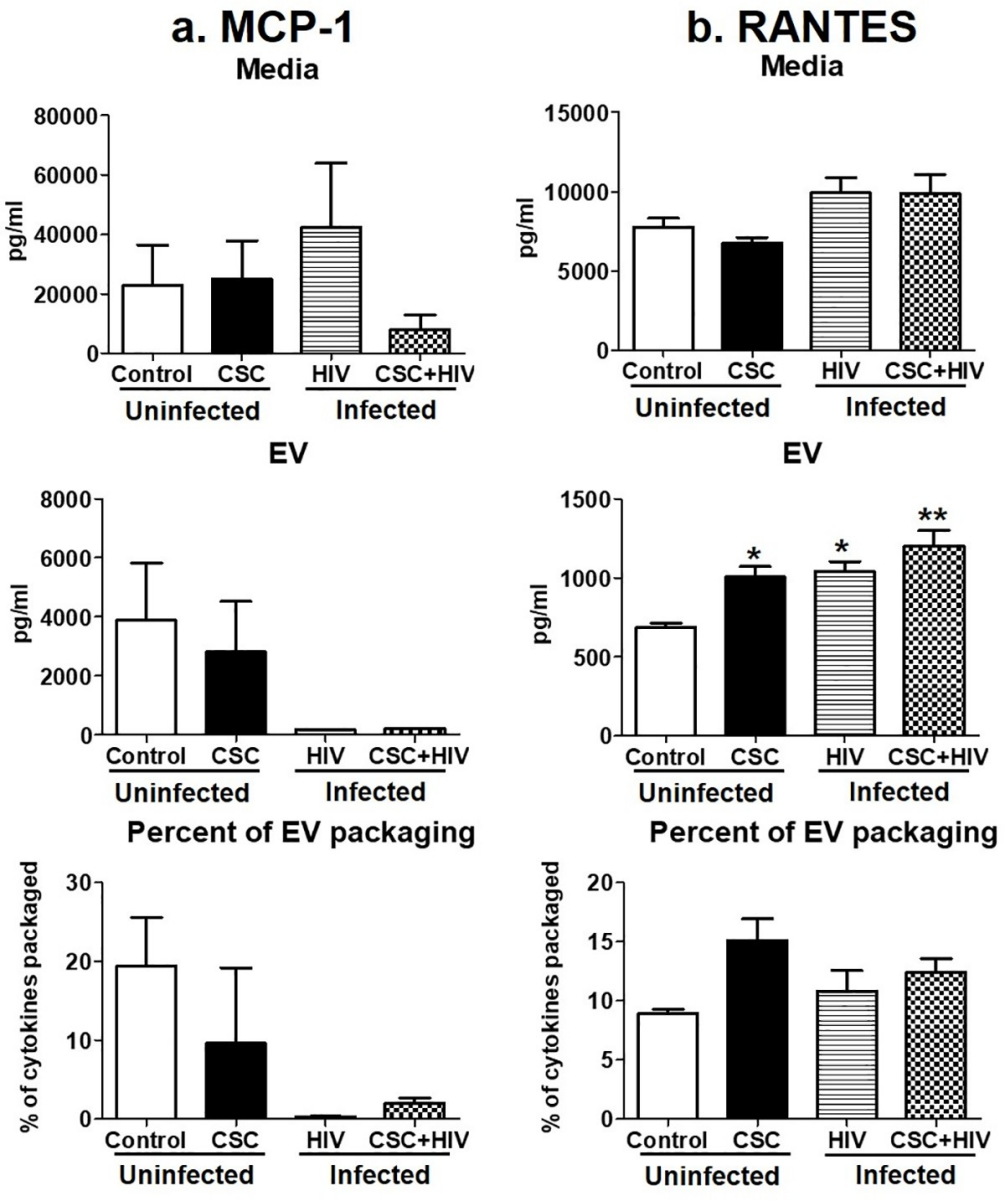
Chemokines in media and Extracellular Vesicles (EV) isolated from U937 (uninfected) and U1 (HIV-infected) macrophages treated with CSC short-term. a. Concentration of MCP-1 in media, EV, and percent of cytokine packaged in EV. Bars represent data from n = 2 replicates. Due to lack of sensitivity, MCP-1 could be measured from two replicates. Therefore, we did not provide any significance value and reported only the change in the trend. b. Concentration of RANTES in media, EV, and percent of cytokine packaged in EV. *p<0.05, **p<0.001 compared to control; #p<0.05, ##p<0.001 compared to HIV. Bars represent data from n = 3 replicates. Statistical analysis performed: One way ANOVA with Tukey’s Multiple comparison test.

MCP-1 and RANTES were the two chemokines measured in this study. The amount of these cytokines varied among all groups, especially MCP-1 (Fig 4A). The amount of RANTES in media was similar among the groups, except after CSC treatment in the uninfected U937 media (Fig 4B). In EVs, the concentration of RANTES gradually increased with CSC treatment, HIV-infection, and with both CSC treatment and HIV infection, compared to control U937 EVs, which was statistically significant in all groups (Fig 4B). This effect was also reflected in the EV packaging. Especially after CSC and CSC + HIV exposure, EV packaging of RANTES was found to be around 15% and 12%, respectively, which was higher compared to control (Fig 4B).

### Effect of long-term CSC exposure on cytokines and chemokines

#### Pro-inflammatory cytokines

**Fig 5:**
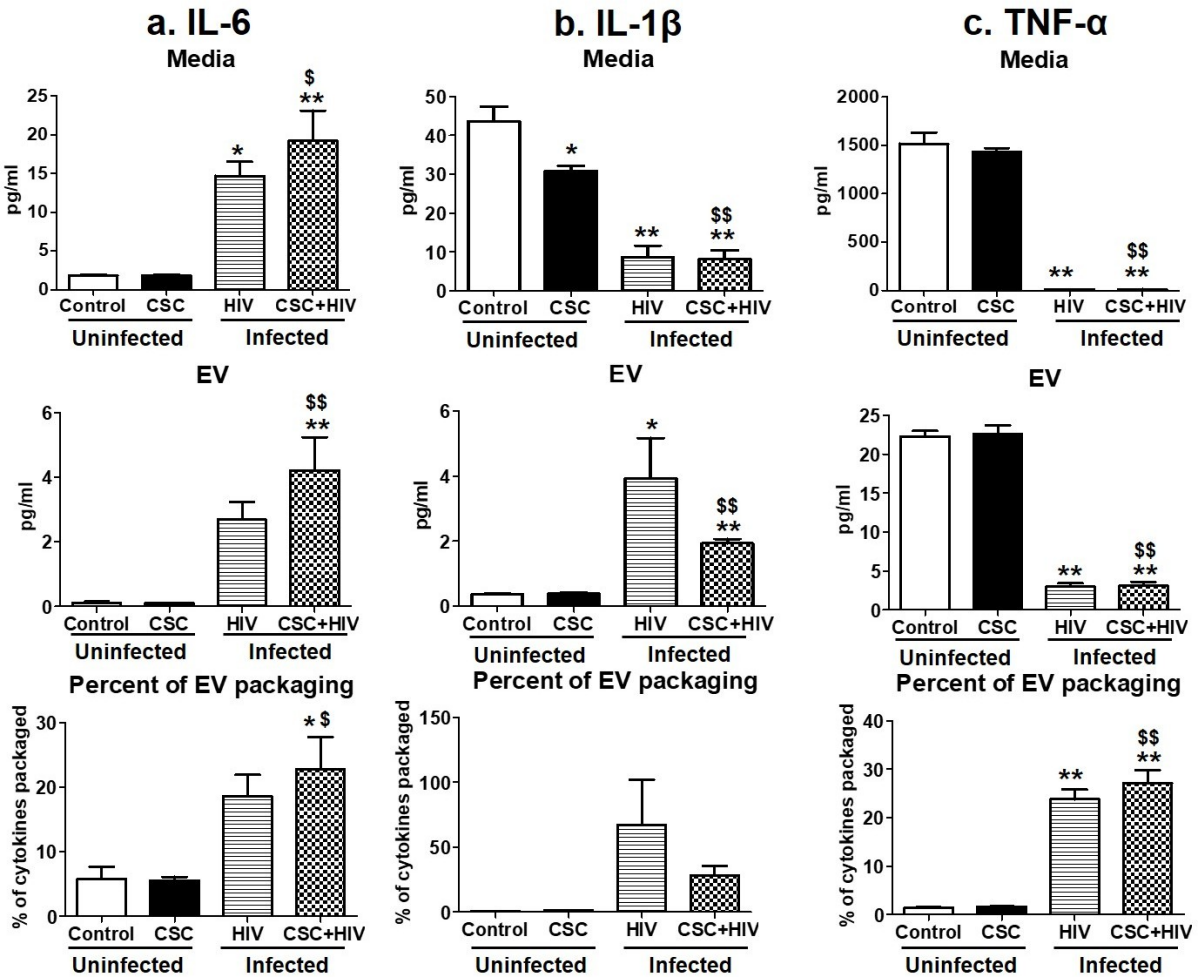
Pro-inflammatory cytokines in media and Extracellular Vesicles (EV) isolated from U937 (uninfected) and U1 (HIV-infected) macrophages treated with CSC long-term. a. Concentration of IL-6 in media, EV, and percent of cytokine packaged in EV. b. Concentration of IL-1β in media, EV, and percent of cytokine packaged in EV. c. Concentration of TNF-α in media, EV, and percent of cytokine packaged in EV. *p<0.05, **p<0.001 compared to control; #p<0.05, ##p<0.001 compared to HIV. $p<0.05 and $ $p<0.001 compared to CSC. Bars represent data from n = 3 replicates. Statistical analysis performed: One-way ANOVA with Tukey’s Multiple comparison test.

The concentration of pro-inflammatory cytokines remained mostly unaltered in media and macrophage-derived EVs from uninfected U937 cells (Fig 5A–5C). IL-1β was significantly lower in the media of uninfected U937 cells compared to control after long-term CSC treatment (Fig 2B). Interestingly, in the presence of CSC+HIV, IL-6 and IL-1β showed more cytokines in EVs from infected U1 cells compared to EVs from uninfected U937 cells (Fig 5A and 5B). Specifically, IL-6 was shown to be significantly high in media and in EVs from HIV-infected U1 cells with or without CSC treatment, compared to control and CSC treatment alone. Approximately 18% and 23% of IL-6 was packaged in EVs from HIV-infected U1 cells and HIV-infected U1 cells upon CSC treatment, respectively (Fig 5A). In the case of IL-1β, media from HIV-infected U1 cells had a lower cytokine concentration compared to media from uninfected U937 cells, whereas EVs from infected U1 cells showed a significantly higher IL-1β concentration (Fig 5B). These results suggest that the cytokines are more driven towards being packaged in EVs upon HIV±CSC exposure. In fact, approximately 67% and 28% of IL-1β was packaged in EVs from HIV-infected U1 cells and HIV-infected U1 cells upon CSC exposure, respectively (Fig 5B). Finally, the amount of TNF-α was higher in the media and in EVs from uninfected U937 cells compared to the media and EVs of HIV-infected U1 cells in the absence or presence of CSC. In terms of EV packaging, 24% and 27% of total TNF-α was packaged within EVs derived from HIV-infected U1 cells and HIV-infected U1 cells upon CSC exposure, respectively (Fig 5C).

#### Anti-inflammatory cytokines

**Fig 6:**
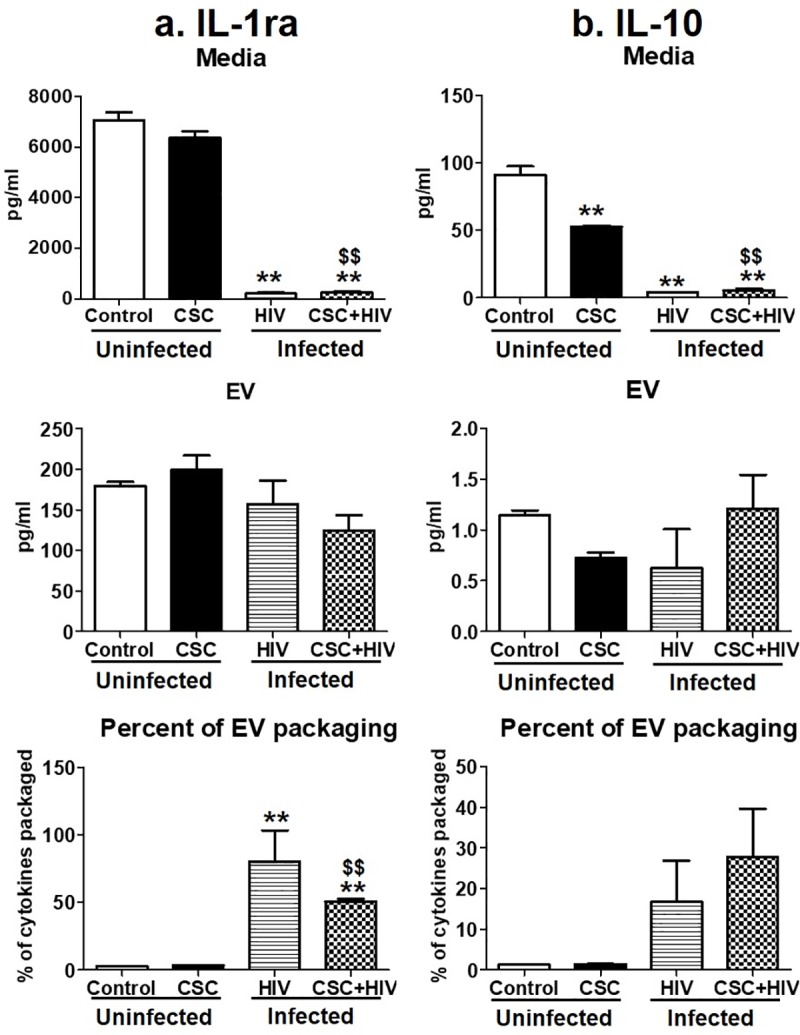
Anti-inflammatory cytokines in media and Extracellular Vesicles (EV) isolated from U937 (uninfected) and U1 (HIV-infected) macrophages treated with CSC long-term. a. Concentration of IL-1ra in media, EV, and percent of cytokine packaged in EV. b. Concentration of IL-10 in media, EV, and percent of cytokine packaged in EV. *p<0.05, **p<0.001 compared to control; #p<0.05, ##p<0.001 compared to HIV. $p<0.05 and $ $p<0.001 compared to CSC. Bars represent data from n = 3 replicates. Statistical analysis performed: One-way ANOVA with Tukey’s Multiple comparison test.

In media, IL-1ra and IL-10 demonstrated a pattern of decreasing cytokine concentration from HIV-infected U1 cells upon CSC exposure, compared to control U937 media (Fig 6A and 6B). CSC treatment reduced the IL-1ra level in EVs from HIV-infected U1 cells with and without CSC exposure compared to control U937 cell-derived EVs. Interestingly, EV packaging of IL-1ra in HIV-infected U1 cells in the absence and presence of CSC exposure was significantly high at 80% and 50%, respectively, explaining the reduction of free cytokines in the media from these groups (Fig 6A). EV packaging of IL-10 increased in HIV-infected U1 cells in the absence and presence of CSC exposure compared to EVs from uninfected U937 cells. This was evident by the amount of cytokines packaged in EVs from HIV-infected U1 cells with and without CSC exposure, which was 16% and 27%, respectively (Fig 6B).

#### Chemokines

**Fig 7:**
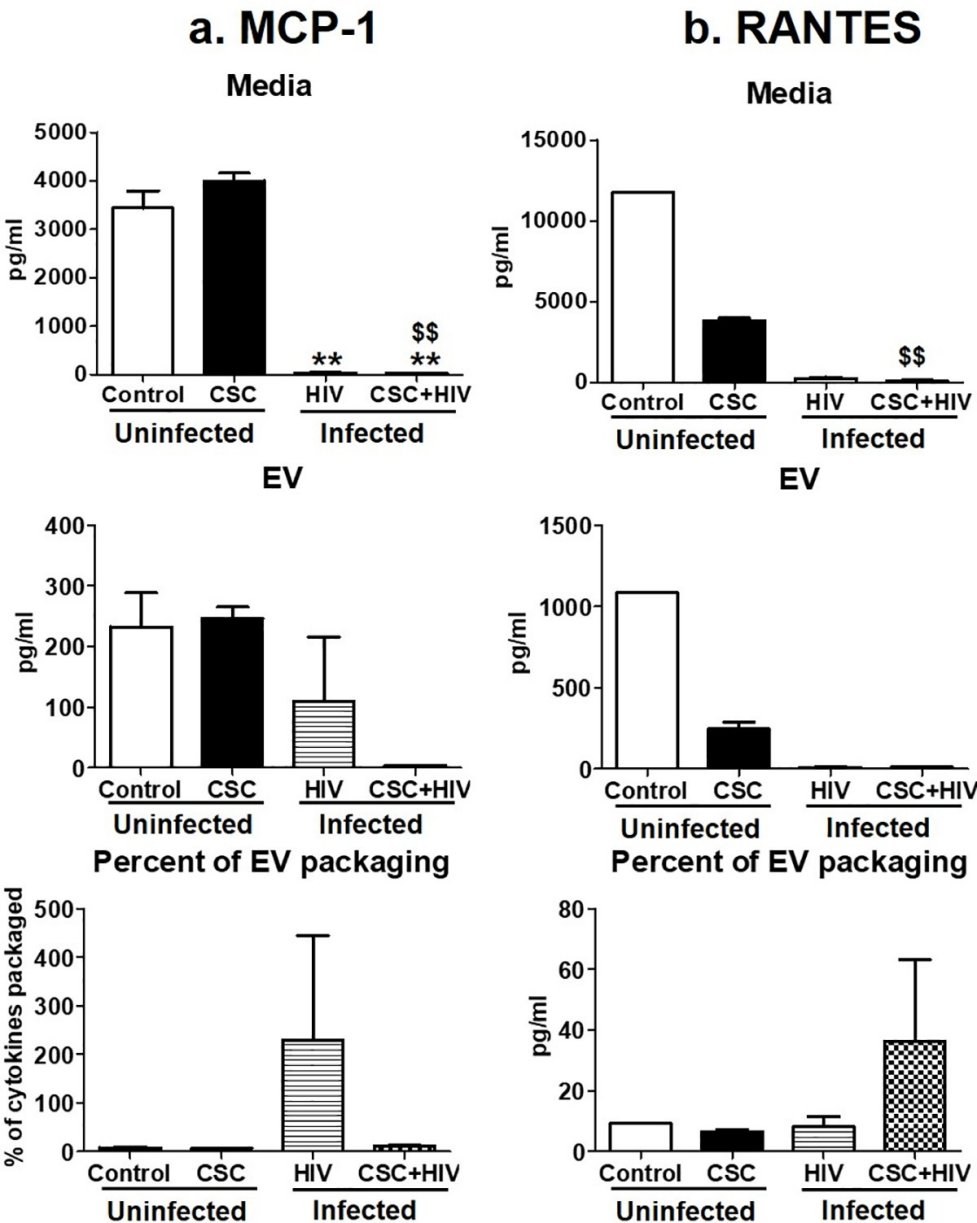
Chemokines in media and Extracellular Vesicles (EV) isolated from U937 (uninfected) and U1 (HIV-infected) macrophages treated with CSC long-term. a. Concentration of MCP-1 in media, EV, and percent of cytokine packaged in EV. Bars represent data from n = 2 replicates. Due to lack of sensitivity, MCP-1 could be measured from two replicates. Therefore, we did not provide any significance value and reported only the change in the trend. b. Concentration of RANTES in media, EV, and percent of cytokine packaged in EV. *p<0.05, **p<0.001 compared to control; #p<0.05, ##p<0.001 compared to HIV. Bars represent data from n = 3 replicates, except control. Statistical analysis performed: One way ANOVA with Tukey’s Multiple comparison test.

Chemokines MCP-1 and RANTES mostly decreased significantly after long-term CSC exposure in HIV-infected U1 macrophage-derived EVs (Fig 7A and 7B). In terms of packaging, MCP-1 was highly driven towards EVs in HIV-infected U1 cells (Fig 7A), whereas RANTES showed a high EV packaging in HIV-infected U1 cells upon CSC treatment (Fig 7B).

### Effect of CSC exposure on EV packaging of CYP enzymes and AOEs

**Fig 8:**
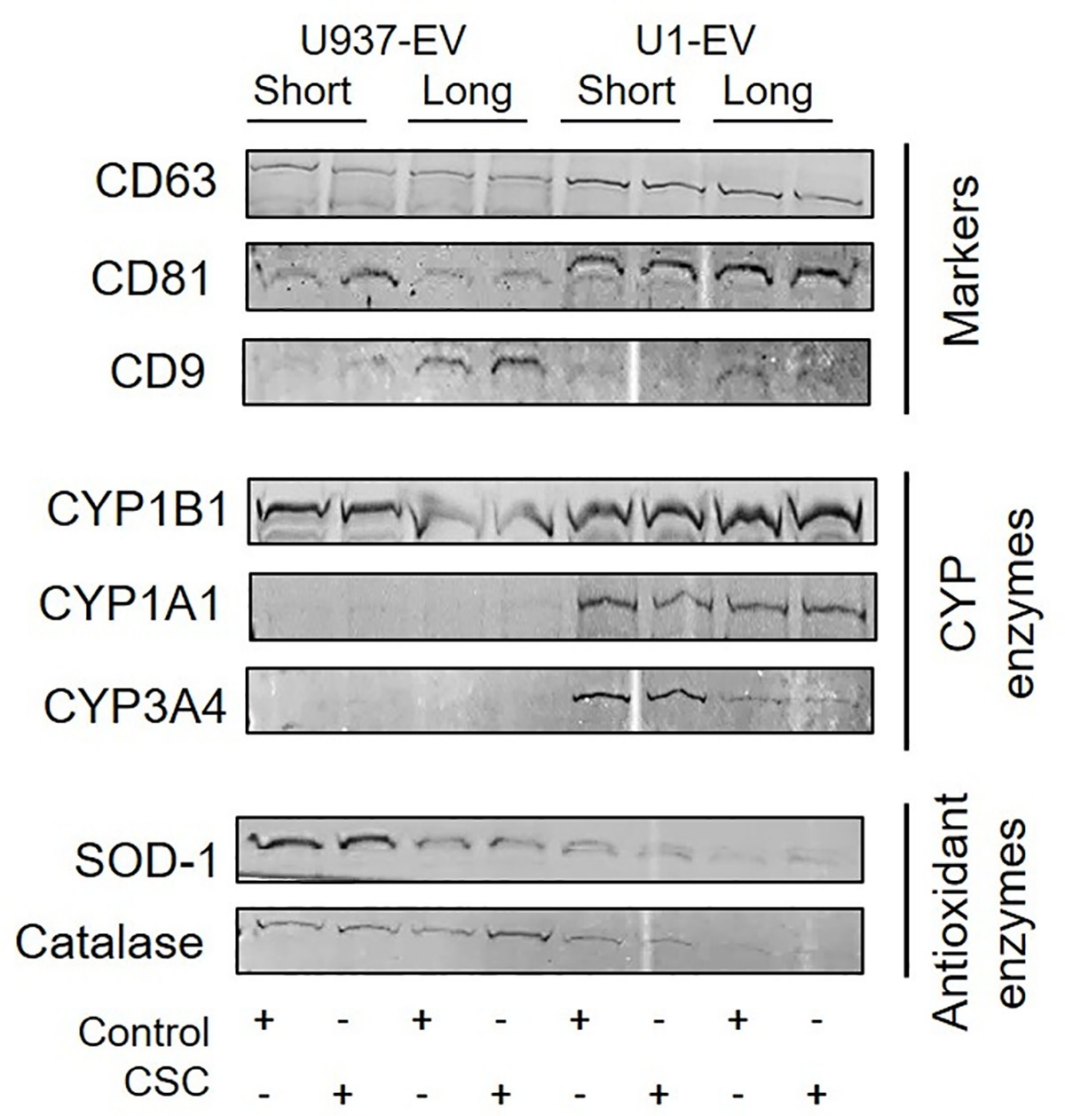
Cytochrome P450 enzymes (CYPs) and Antioxidant enzymes (AOEs) in Extracellular Vesicles (EV) isolated from U937 (uninfected) and U1 (HIV-infected) macrophages treated with CSC for short-term and long-term durations. Markers for extracellular vesicles namely: CD63, CD81, and CD9 were present in EV. Protein expression levels of CYP enzymes such as: CYP1B1, CYP1A1, and CYP3A4; AOEs such as SOD-1 and catalase were checked. This is a representative blot of at least two experiments. Equal amounts of protein were loaded in each well.

CYP enzymes, which are responsible for the metabolism of smoking constituents and subsequent induction of oxidative stress, as well as AOEs, were measured in EVs derived from HIV-infected U1 and uninfected U937 macrophages exposed to CSC for short- and long-term durations by western blot (Fig 8, for full blot view, please refer to S1 Fig). We loaded equal amounts of protein in each lane of the gel. We observed a slight increase in the expression of marker proteins, namely CD63 and CD81, in the HIV-infected U1 macrophage-derived EVs, compared to the EVs derived from uninfected U937 cells. Although these marker proteins are known to vary under different conditions, it is also possible that this increase is as a result of increased number and/or protein levels of EVs in U1 cells compared to U937 cells. CD9 also showed a variation in expression in EVs derived from HIV-infected U1 cells. This is not surprising, as these marker proteins are not traditionally considered to be housekeeping proteins, and they are shown to be altered in EVs depending upon EV origin and the conditions of the parent cells [27]. We measured the expression levels of CYP enzymes 1A1, 1B1, 3A4 (polyaryl hydrocarbon metabolizer and major drug metabolizing enzymes- [21, 28–31]) in uninfected U937 and HIV-infected U1 macrophage-derived EVs with or without CSC exposure. Although there appears to be a slight increase in the expression of CYP1B1 in HIV-infected macrophage-derived EVs, CYPs 1A1 and 3A4 were clearly undetectable in EVs derived from uninfected U937 cells but were present in EVs derived from HIV-infected U1 cells. Finally, we also measured common anti-oxidant enzymes (AOEs) such as SOD-1 and catalase. There was a clear decrease in the expression of both enzymes in EVs isolated from HIV-infected U1 cells compared to EVs derived from uninfected U937 cells. Our data suggest that EVs derived from HIV-infected U1 cells demonstrate a higher CYP expression and lower AOE expression, which together indicate an overall increase in oxidative stress-inducing elements in EVs derived from HIV-infected U1 cells. However, in all cases, CSC did not alter the expression level of either CYPs or AOEs in EVs from uninfected U937 or HIV-infected infected U1 cells.

## Discussion

Extracellular vesicles and the potential role of their cargos have been vastly studied in several disease conditions; however, the understanding of their contribution to HIV pathogenesis is still largely unknown. We have previously demonstrated that macrophage-derived EVs, in the presence of CSC and HIV, can either protect or damage cells depending on the origin [20]. In this study, we characterized EVs obtained from uninfected U937 and HIV-infected U1 cells upon CSC exposure. We then examined the differential packaging of cytokines, chemokines, CYPs, and AOEs within EVs upon exposure to smoking constituents in HIV-infected U1 and uninfected U937 macrophages. Overall, we found increased packaging of pro-inflammatory cytokines and oxidative stress-inducing CYP enzymes, together with a decreased packaging of anti-inflammatory cytokines and AOEs, in EVs derived from CSC-exposed, HIV-infected U1 compared to uninfected U937 macrophages. Our findings suggest that HIV, in the presence of CSC, can enhance the packaging of inflammatory and oxidative stress modulators in EVs, which may potentially exacerbate smoking-mediated HIV pathogenesis. This is the first study to investigate the packaging of inflammatory and oxidative stress modulators in macrophage-derived EVs, particularly upon exposure to CSC, in the absence and presence of HIV.

**Fig 9:**
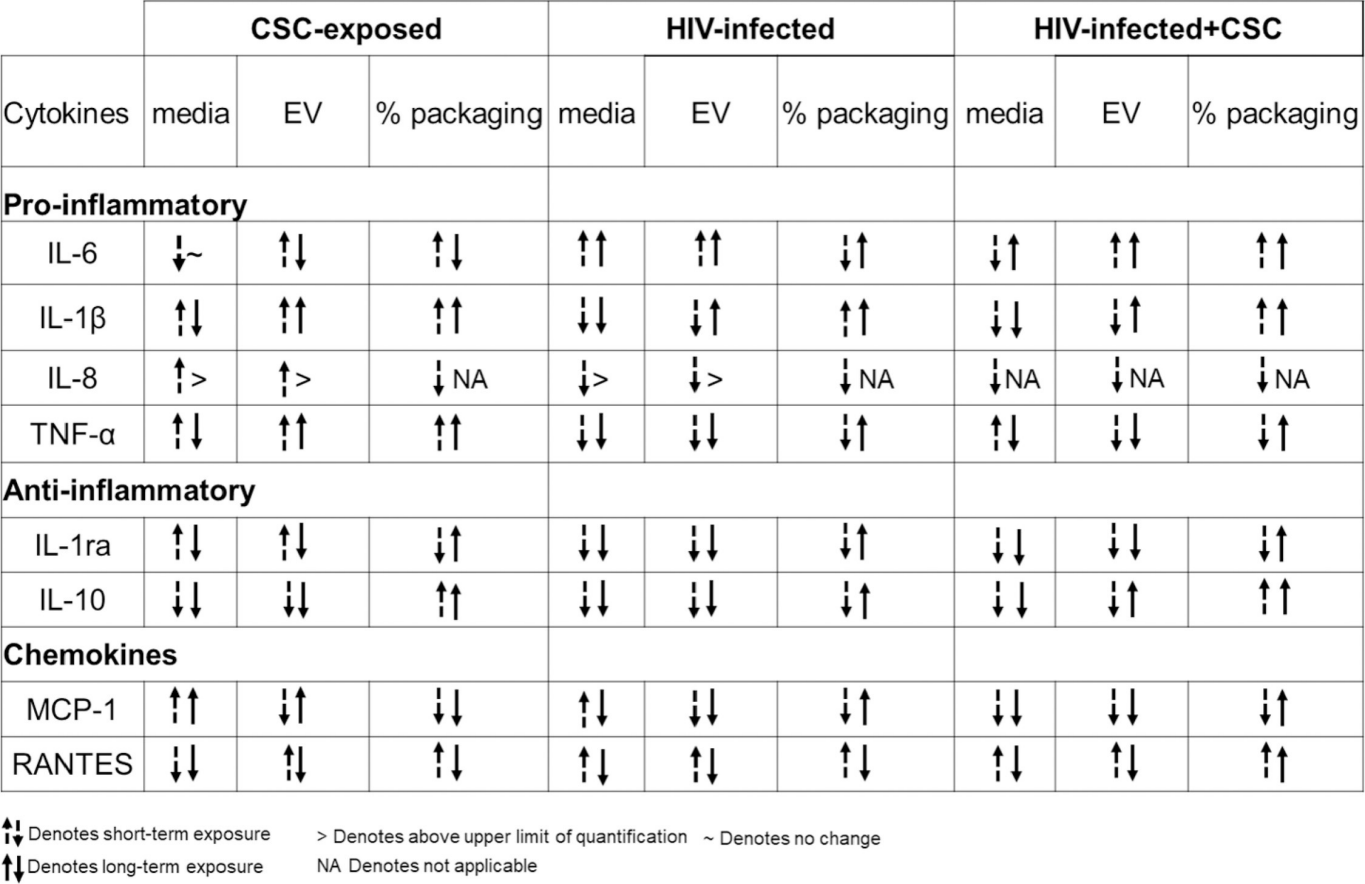
Comparison between the short-term vs. long-term treatment of CSC ± HIV on EVs derived from uninfected U937 and HIV-infected U1 macrophages.

Cigarette smoking is strongly associated with inflammatory responses, demonstrated by alterations in cytokine expressions, which vary widely among population groups based on age, smoking status, and other associated conditions [32]. We have previously demonstrated the differential packaging of cytokines in plasma-derived EVs from HIV-infected and uninfected smokers [4]. Similarly, the current study also demonstrates alterations in the cytokine and chemokine levels of macrophage-derived EVs under the influence of CSC. Moreover, CSC primarily induced the expression of pro-inflammatory cytokines, both in the media and in EVs derived from macrophages. IL-6, in particular, displayed a noticeably high percentage of EV packaging (Figs 2A and 5A). Our finding is also supported by previous studies indicating that IL-6 is increased in smokers [33]. Further, the mixed response we observed regarding anti-inflammatory cytokines, both in the media and in EVs, is also supported by literatures. In fact, nicotine is known to have a conflicting impact on cytokines [34–36]. As for chemokines, both MCP-1 and RANTES demonstrated opposite trends in packaging after short- and long-term exposures (Fig 9). This suggests that the duration of smoking constituent exposure may impact chemokine levels.

HIV is known to be associated with a significant increase in IL-6 in the presence of cigarette smoking and other comorbid conditions, even in patients who are on anti-retroviral therapy (ART) [37–41]. EVs derived from HIV-infected U1 macrophages are shown to exacerbate cytotoxicity and HIV replication in recipient cells [20]. Moreover, the EV contents vary greatly according to the stages of viral infection [42, 43]. Consistent with previous studies, the current study shows a general upward trend in the packaging of cytokines, especially pro-inflammatory cytokines IL-6, Il-1β, and TNF-α, as well as the chemokine RANTES, upon HIV exposure (Fig 9). RANTES, along with other chemokines, is known to potentially upregulate HIV replication in macrophages by recruiting target cells [44]. Thus, our finding suggests that RANTES, produced within cells, is transported to other cells via EVs, further contributing to HIV replication. A decrease in the secretion of anti-inflammatory cytokines, especially IL-10, in the media following CSC and HIV exposures, conforms to the role of IL-10 as an HIV-immunosuppressive agent [45]. In contrast, we observed an increase in the EV packaging of anti-inflammatory cytokines, including IL-10, upon exposures to both CSC and HIV together (Fig 9). This trend is similar to a previously observed trend in plasma-derived EVs from HIV-infected smokers, where IL-10 was completely packaged in EVs [4]. This finding suggests a potential role of EVs in transmitting protective elements to recipient cells. In fact, our previous study also demonstrated that EVs from monocytes could be protective against cytotoxicity and HIV replication in recipient macrophages [20]. However, the protective influence of EVs is mostly limited to the earlier stages of HIV infection.

Induction of cytokines and chemokines is critical during the early and later stages of HIV infection [46, 47]. Consistent with these findings, our current study illustrates a higher packaging of cytokines and chemokines within EVs following long-term exposure to CSC than after short-term exposure, both in the absence and in presence of HIV infection. Moreover, IL-6 packaging demonstrated an additive effect after long-term CSC exposure in the presence of HIV-infection. In addition, our study suggests that smoking constituents further enhance TNF-α in EVs derived from HIV-infected cells. This observation corroborates other studies suggesting the role of the exosomal pro-TNF-α enzyme, which may potentially contribute to the exacerbation of HIV replication [48]. Further, the EV packaging of both anti-inflammatory cytokines, IL-1ra and IL-10, after long-term CSC exposure in the presence of HIV, was noticeably high when compared to the control, as well as when compared to short-term exposures under similar conditions (Figs 3 and 6). This suggests that long-term exposures of cigarette smoke to HIV-infected macrophages potentially trigger relatively high expression of cytokine packaging in EVs regardless of whether the cytokines are pro- or anti-inflammatory in nature. Consistent with previous reports, the elevated level of MCP-1 in both media and EVs upon long-term CSC exposure suggests increased recruitment of monocytes/macrophages to the site of active inflammation [49]. Further, an increase in RANTES EV packaging upon CSC and HIV exposures together, is consistent with a similar trend observed in an *ex vivo* system, in which the RANTES level is elevated in HIV-infected smokers irrespective of ART use [3]. Moreover, our previous *ex-vivo* study demonstrates a similar upward trend in the EV packaging of IL-8 and RANTES, with a downward trend for IL-1ra and MCP-1 [4].

Smoking/CSC is known to induce oxidative stress via the NF-κβ pathway [15, 50]. Literatures have also established that smoking constituents/CSC induce oxidative stress via inducing CYPs, especially CYP2A6 and 1A1, which in turn metabolize smoking constituents in macrophages [12, 14, 51, 52]. However, the lack of increased CYP packaging in EVs upon CSC exposure suggests that despite enhanced cellular expression of CYPs upon CSC exposure, CYP packaging in EVs is limited. On the other hand, a higher packaging of CYP1A1, concurrent with a lower packaging of SOD1 and catalase in EVs upon HIV exposure, suggests an overall increase in the packaging of oxidative stress factors in EVs. The finding is somewhat consistent with our own *ex vivo* finding in which there was an increase in the cellular expression of CYPs in monocytes from HIV-infected subjects [21]. Overall, this finding suggests a role for EVs in exacerbating HIV pathogenesis via oxidative stress pathways (Fig 8).

**Fig 10:**
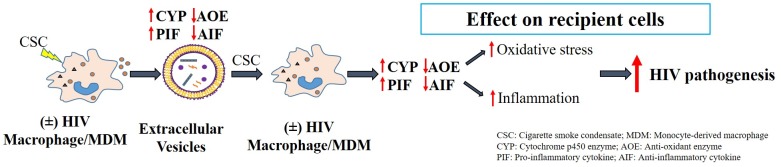
Proposed mechanistic pathway for the effect of differential packaging of inflammatory and oxidative stress modulators in macrophage-derived extracellular vesicles in exacerbating HIV pathogenesis. Macrophages release extracellular vesicles, and upon exposure to CSC in the absence and presence of HIV, the contents of these EVs are modified. Overall, pro-inflammatory cytokines and CYP enzymes demonstrated higher packaging in EV whereas, anti-inflammatory cytokines and anti-oxidant enzymes showed a decreasing pattern in EV packaging. This subsequently leads to induction of oxidative stress and inflammation, ultimately exacerbating HIV pathogenesis.

In addition to delivering cargo to neighboring and distant cells, EVs, particularly macrophage-derived EVs, have the advantage of penetrating the highly selective, semipermeable blood brain barrier (BBB) and deliver cargo to the resident cells [53]. This EV characteristic is important in studying HIV-associated neurocognitive disorders (HAND) [54]. Moreover, smoking constituents are known to cause damage to the BBB and make it more permeable to the CNS [55]. Thus, it is possible that EVs packaged with oxidative stress (CYPs and AOEs) and inflammatory elements, especially IL-6, would further enhance oxidative stress, inflammation, and subsequently HIV pathogenesis in the CNS, eventually leading to HAND in HIV-smokers. However, the quantity of oxidative stress and inflammatory elements packaged within EVs is rather small. Fig 10 represents a proposed mechanistic pathway demonstrating the potential role of differential packaging of inflammatory and oxidative modulators in EVs in HIV pathogenesis. Our earlier studies have demonstrated the impact of EVs in recipient cells [19, 20]. To ascertain whether these EV contents are physiologically relevant, further investigations are warranted. In addition, identifying the specific role of EVs containing cytokines/chemokines, especially IL-6, and EVs containing CYPs and AOEs, as well as other EV factors, e.g. proteins, mRNA and miRNA, on HIV pathogenesis and HAND, is highly desirable.

## Supporting information

S1 Fig Western blot of exosomal marker proteins (a) CD63, CD81, CD9; Cytochrome p450 enzymes (b) 1B1, 1A1, 3A4; Antioxidant enzymes (c) SOD-1, catalase. Each band is presented as whole blot.
(DOCX)
