# Supplementary material for: Differential packaging of inflammatory cytokines/ chemokines and oxidative stress modulators in U937 and U1 macrophages-derived extracellular vesicles upon exposure to tobacco constituents
Source: PLoS One. 2020 May 20;15(5):e0233054. doi: 10.1371/journal.pone.0233054 (PMC7239484; doi:10.1371/journal.pone.0233054)
Supplement: S1 Fig — Western blot of exosomal marker proteins (a) CD63, CD81, CD9; Cytochrome p450 enzymes (b) 1B1, 1A1, 3A4; Antioxidant enzymes (c) SOD-1, catalase. Each band is presented as whole blot. (DOCX) [file pone.0233054.s001.docx]

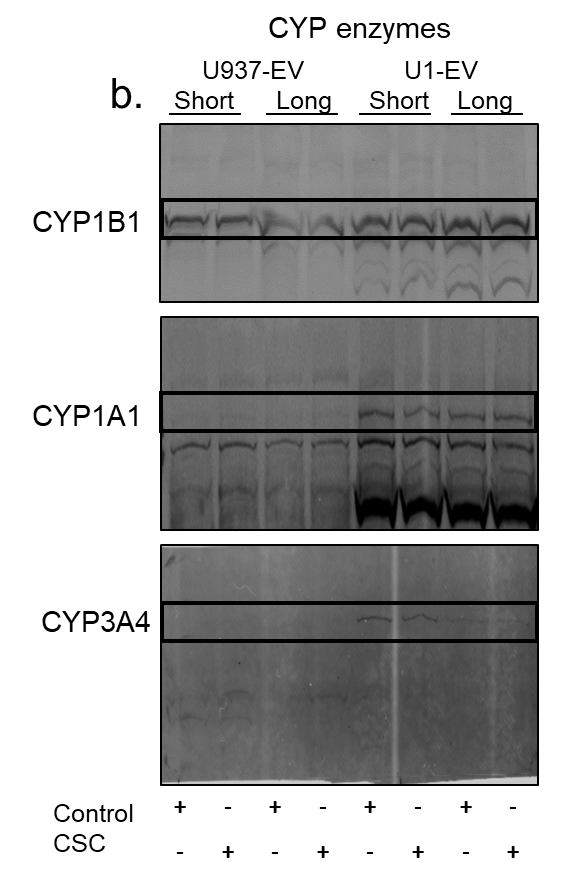

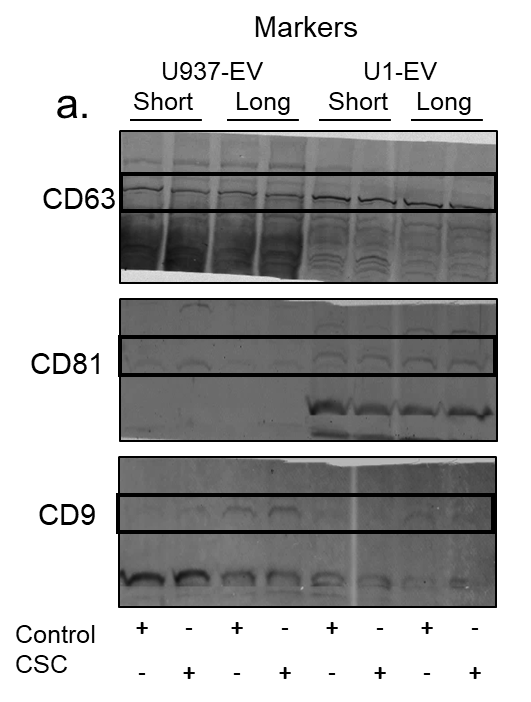


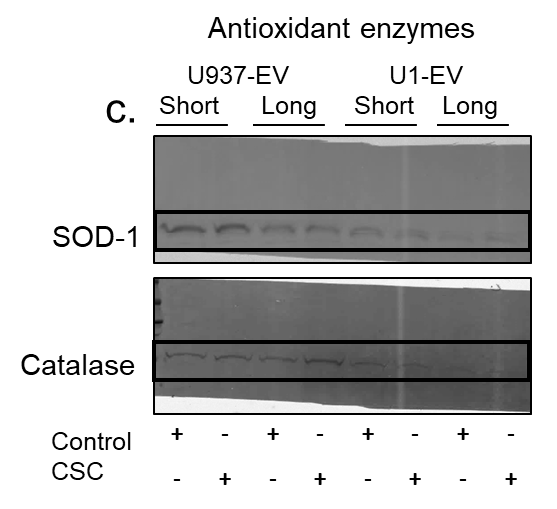


**Supplementary figure 1**: Western blot of exosomal marker proteins (a) CD63, CD81, CD9; Cytochrome p450 enzymes (b) 1B1, 1A1, 3A4; Antioxidant enzymes (c) SOD-1, catalase. Each band is presented as whole blot.
